# Fat and Sugar—A Dangerous Duet. A Comparative Review on Metabolic Remodeling in Rodent Models of Nonalcoholic Fatty Liver Disease

**DOI:** 10.3390/nu11122871

**Published:** 2019-11-24

**Authors:** Ines C.M. Simoes, Justyna Janikiewicz, Judith Bauer, Agnieszka Karkucinska-Wieckowska, Piotr Kalinowski, Agnieszka Dobrzyń, Andrzej Wolski, Maciej Pronicki, Krzysztof Zieniewicz, Paweł Dobrzyń, Marcin Krawczyk, Hans Zischka, Mariusz R. Wieckowski, Yaiza Potes

**Affiliations:** 1 Nencki Institute of Experimental Biology of Polish Academy of Sciences, 02-093 Warsaw, Polandj.janikiewicz@nencki.gov.pl (J.J.); a.dobrzyn@nencki.gov.pl (A.D.); p.dobrzyn@nencki.gov.pl (P.D.); y.potes-ochoa@nencki.edu.pl (Y.P.); 2 Institute of Toxicology and Environmental Hygiene, Technical University Munich, School of Medicine, Biedersteiner Strasse 29, D-80802 Munich, Germany; u.bauer@tum.de (J.B.); zischka@helmholtz-muenchen.de (H.Z.); 3 Department of Pathology, The Children’s Memorial Health Institute, 04-730 Warsaw, Poland; A.Karkucinska-Wieckowska@ipczd.pl (A.K.-W.); m.pronicki@ipczd.pl (M.P.); 4 Department of General, Transplant and Liver Surgery, Medical University of Warsaw, 02-091 Warsaw, Poland; piotr.kalinowski@wum.edu.pl (P.K.); krzysztof.zieniewicz@wum.edu.pl (K.Z.); 5 Department of Interventional Radiology and Neuroradiology, Medical University of Lublin, 20-090 Lublin, Poland; andrzej.s.wolski@gmail.com; 6 Laboratory of Metabolic Liver Diseases, Department of General, Transplant and Liver Surgery, Centre for Preclinical Research, Medical University of Warsaw, 02-091 Warsaw, Poland; Marcin.Krawczyk@uks.eu; 7 Department of Medicine II, Saarland University Medical Center, 66421 Homburg, Germany; 8 Institute of Molecular Toxicology and Pharmacology, Helmholtz Center Munich, German Research Center for Environmental Health, Ingolstaedter Landstrasse 1, D-85764 Neuherberg, Germany

**Keywords:** mitochondria, NAFLD-inducing diets, nonalcoholic fatty liver disease, oxidative stress

## Abstract

Nonalcoholic fatty liver disease (NAFLD) is a common disease in Western society and ranges from steatosis to steatohepatitis to end-stage liver disease such as cirrhosis and hepatocellular carcinoma. The molecular mechanisms that are involved in the progression of steatosis to more severe liver damage in patients are not fully understood. A deeper investigation of NAFLD pathogenesis is possible due to the many different animal models developed recently. In this review, we present a comparative overview of the most common dietary NAFLD rodent models with respect to their metabolic phenotype and morphological manifestation. Moreover, we describe similarities and controversies concerning the effect of NAFLD-inducing diets on mitochondria as well as mitochondria-derived oxidative stress in the progression of NAFLD.

## 1. Introduction

Nonalcoholic fatty liver (NAFL) is characterized by an increased accumulation of lipids in the liver [1], and according to current estimations, it affects 25% of adults worldwide; however, this number is predicted to increase rapidly in the coming years [2]. This high incidence is mainly due to the increasing prevalence of exogenous risk factors for nonalcoholic fatty liver disease (NAFLD), e.g., overweight and obesity. Although the prevalence of NAFLD in pediatric populations is also variable, it correlates with BMI, and according to a meta-analysis by Anderson et al., it has reached 34.2% in clinically obese populations [3]. NAFLD triggers are further modulated by an inherited predisposition for NAFLD [4]. Interestingly, obesity does not represent *conditio sine qua non* for the development of NAFLD. Progressive liver steatosis eventually leads to liver cirrhosis and hepatic decompensation necessitating transplantation; however, we still lack clinical markers that can predict the speed of progressive NAFLD in the clinical setting. In particular, patients with the severe form of fatty liver, namely, nonalcoholic steatohepatitis (NASH), are at risk of progressive liver disease.

## 2. NAFLD—From the Patient’s and Doctor’s Points of View 

### 2.1. Pathogenesis of NAFLD

The pathogenesis of NAFLD is closely related to mechanisms governing the development of obesity and metabolic syndrome, of which NAFLD is considered one of the main components [5]. At the systemic level, there is impaired control of food intake resulting in hyperalimentation, intestinal dysbiosis leading to gut dysfunction [6], insulin resistance [7], abnormal adipokine [8] and gastrointestinal hormone secretion [9] and activation of proinflammatory factors [10]. As the disease progresses from simple steatosis to advanced NASH, these mechanisms weigh differently in the pathological processes and resulting injury to the hepatic tissue. The pathogenesis of NAFLD is commonly explained by a two-hit theory, but for a more accurate description, multiple interacting factors should be considered [11,12]. The ‘first hit’ is the development of ‘simple’ hepatic steatosis represented by accumulation of triacylglycerol (TAG) droplets in hepatocytes. The ’second hit’ in pathogenesis is related to oxidative injury but involves a multifactorial process that includes an exacerbation of mechanisms related to insulin resistance and increasing oxidative stress, lipid peroxidation, endoplasmic reticulum stress and inflammation. The genetic predisposition has also been grossly implicated in the pathogenesis and progression of NAFLD [13]. Performed genetic studies have shown that carriers of the risk alleles in numerous genes (e.g., *PNPLA3* [14], *MBOAT7* [15] or *TM6SF2* [16]) are at-risk of increased hepatic lipid accumulation; however, the exact mechanisms of NAFLD in the setting of inherited predisposion have not been fully elucidated. 

The mechanisms of hepatic TAG accumulation include increased inflow of fatty acids (FAs) to the liver from circulation, hepatocyte de novo synthesis and impaired clearance via β-oxidation or secretion from hepatocytes as very low-density lipoproteins (VLDL). Increased delivery from circulation is the primary source of hepatic FAs followed by de novo lipogenesis [17]. High levels of circulating FAs are a consequence of both increased lipolysis in adipose tissue and dietary intake. Adipose tissue does not only contribute to NAFLD as a source of FA; adipocytes secrete adipokines that have protective effects against NAFLD, such as adiponectin and visfatin, as well as resistin and leptin, which contribute to hepatic steatosis and insulin resistance. Under physiologic conditions, adiponectin regulates insulin sensitivity and fatty acid oxidation, leading to decreased lipid accumulation. In NAFLD, the secretion of adipokines shifts to an abnormal pattern with a decreased secretion of adiponectin [18] and an increase in the levels of resistin, leptin and proinflammatory cytokines such as tumor necrosis factor alpha (TNF-α), interleukins (IL-1, IL-6) and monocyte chemoattractant protein-1 (MCP-1), which promote hepatic insulin resistance [19]. At the hepatic level, the activation of proinflammatory mechanisms involves secretion of the proinflammatory cytokines TNFα, IL-6, and IL-1β by Kupffer cells and macrophages. This, in turn, leads to activation of hepatic stellate cells, which is a pivotal step in the development of fibrosis. Increased inflammatory conditions further exacerbate insulin resistance both in adipose tissue and the liver.

Patients with NAFLD not only are at risk of progressive liver disease but also have a substantially increased risk of cardiovascular diseases and death due to malignancies [20]. Data obtained from a cohort of 229 biopsy-proven NAFLD patients who were followed up for a mean 26.4 (range 6–33) years showed that the presence of fatty liver is associated with an increased risk of cardiovascular disease (HR 1.55, *p* = 0.01), liver cancer (HR 6.55, *p*  = 0.001), infectious diseases (HR 2.71, *p*  = 0.046), and liver cirrhosis (HR 3.2, *p*  = 0.041) [21]. Even before hepatic decompensation occurs, patients with NAFLD suffer from impaired kidney function [22] and an increased incidence of cardiovascular diseases [23]. Hence, NAFL should be addressed clinically as a systemic disease. To date, however, pharmacological therapies for NAFLD are lacking [24], while hypocaloric diet, physical activity and “healthy lifestyle” remain the cornerstones of the therapy for patients with fatty liver [25]. An interventional approach, including metabolic surgery and endoscopic procedures, may be beneficial for selected groups of patients, e.g., morbidly obese patients with NAFLD [26,27]. Patients with NASH-related liver cirrhosis and end-stage liver disease or cirrhosis with hepatocellular carcinoma are increasingly becoming candidates for liver transplantation [28]. 

### 2.2. Diagnosis

To date, liver biopsy represents the only available method that allows discrimination between NAFL and NASH [29]. However, this procedure is invasive and is potentially associated with complications. Hence, noninvasive methods for quantifying liver injury have been developed. Abdominal sonography is commonly available and is a sensitive and specific tool allowing detection of hepatic steatosis (patients with NAFLD present with “bright liver”), however, this method fails to distinguish NASH from simple steatosis. (“bright liver”); however, this method fails to detect NASH. Vibration-controlled transient elastography (VCTE) with controlled attenuation parameter (CAP) allows parallel noninvasive measurements of liver fibrosis and steatosis and can be considered for patients with NAFLD as a follow-up tool [30]. This approach can be combined with serum-based markers of liver scarring to further increase the accuracy in diagnosing liver fibrosis [31]. Indeed, the exact quantification of liver scarring represents the central point in diagnosing patients with NAFLD since numerous studies have shown that fibrosis represents the limiting factor in patient survival [32,33]. Diagnosis of steatohepatitis in patients is based on histopathological evaluation of liver biopsies showing characteristic morphological features: macrovesicular steatosis, ballooned hepatocytes with cytoplasmic inclusions (Mallory–Denk bodies (MDBs)) and lobular inflammation. Furthermore, hepatocyte necrosis or apoptosis, as well as cholangiolar changes (e.g., bilirubinostasis and ductular reaction), can be observed. The degree of fibrosis depends on the progression status of the disease and may range from pericellular fibrosis of single hepatocytes via incomplete fibrous septa and bridging septal fibrosis up to complete liver cirrhosis. In the case of NAFLD/NASH, these changes represent the hepatic manifestation of metabolic syndrome.

## 3. A Round Trip of NAFLD and Diabetes: Two Entities in a Bidirectional Relationship

The epidemiology of NAFLD already parallels the pandemic of obesity. In a disturbed metabolic milieu, both conditions are interrelated with type 2 diabetes (T2D) by common pathophysiological mechanisms—insulin resistance and the progression of compensatory hyperinsulinemia to β-cell demise or defective lipid metabolism [34,35]. This relationship remains complex and bidirectional, and as such, NAFLD may predispose individuals to T2D but not surprisingly, obesity-driven T2D promotes NASH and advanced liver fibrosis, suggesting that end-stage liver disease should be considered an overlooked complication of diabetes. From the diabetological point of view, NAFLD and T2D have become a dangerous disease combination due to an increased insulin demand, aggravated insulin resistance, microvascular complications, and hyperglycemia that is increasingly difficult to control [36].

### 3.1. From NAFLD to T2D

The new paradigm suggests that during NAFLD-driven metabolic imbalance, noncirrhotic, prefibrotic NAFLD predisposes the development of incident T2D before more advanced stages of liver fibrosis occur [37]. Ultimately, several epidemiological studies have consistently showed NAFLD to be an independent risk factor for the development of T2D, irrespective of the method used to assess NAFLD-liver biopsy, ultrasound imaging, serum liver enzymes or radiological evidence [37]. Fairly recently, three systematic reviews of population-based studies showed that both fibrosis score-proven, ultrasonography-confirmed and imaging-diagnosed NAFLD were associated with a twofold increased risk of developing diabetes over a median period of 5 years after adjusting for confounders and known risk factors for T2D [38,39,40]. Moreover, diagnosed NAFLD predicts the occurrence of prediabetes and the development of glucose intolerance [41]. In a Korean cohort of prediabetic individuals with NAFLD and impaired fasting glucose, the incidence of T2D at the 5-year follow-up was accelerated by almost 9-fold [42]. The Fatty Liver Index (FLI), which is a surrogate marker of hepatic steatosis and correlates with insulin resistance, was identified as a potent predictor for the conversion to new-onset diabetes in Spanish and French patients with prediabetes ([43] and [44], respectively).

Apart from insulin resistance, similar to prediabetes and T2D, the global burden of NAFLD depends on obesity [45]; however, lean individuals with NAFLD possess a distinct clinical profile in comparison to overweight–obese NAFLD patients [46]. Nonobese individuals with NAFLD showed hallmarks of insulin resistance and impaired fasting glucose [47,48]. The prevalence of NAFLD in lean individuals reached 7%–9% in the American population and was independently associated with a decreased likelihood of having insulin resistance [46,49]. Moreover, the incidence rate of T2D in the nonoverweight group with NAFLD was 14.4% and 6.7% in Japanese individuals and in the NHANES III cohort, respectively [50,51].

During almost 4 years of follow-up, cross-sectional studies within Korean and Chinese populations revealed an independent association between NAFLD severity and an increased incidence of T2D, regardless of the euglycemic range of Hb1Ac or glucose [52,53]. Notwithstanding limitations of the current techniques implemented to diagnose NAFLD, more advanced NAFLD carries a greater risk of T2D with the passing of time.

### 3.2. Incidence of NAFLD in T2D

Recent meta-analysis and cohort studies revealed a 50%–69% prevalence of either liver magnetic resonance spectroscopy (^1^H-MRS)- or ultrasound-confirmed NAFLD among type 2 diabetic individuals [54,55,56,57]. Higher plasma HbA1c levels were interrelated with elevated NAFLD prevalence. T2D patients with NAFLD suffered severe systemic (liver/muscle) and adipose tissue insulin resistance in comparison to nonobese individuals without NAFLD [56]. Approximately 80% of nonobese subjects without NAFLD sustain normal levels of plasma aminotransferases [36,56]. Nevertheless, no association between the incidence of NAFLD and glycemic control or degenerative diabetic complications was confirmed, indicating that NAFLD in diabetic patients may develop and progress independently of diabetes progression itself [57]. In contrast, patients with T2D complicated by NAFLD remain at an increased risk of NASH, advanced fibrosis, cirrhosis and hepatocellular carcinoma in the background of T2D [58,59,60]. Moreover, either improvement or transient remission of NAFLD were positively associated with reduced T2D incidence and may lead to improvement of glucose tolerance [61,62]. A change in NAFLD status over time and a reduction in hepatic fat accumulation could prevent future T2D development regardless of BMI change [63]. On the other hand, obesity and insulin resistance drive fatty infiltration of not only the liver but also, the pancreas. Based on autopsies from clinical patients, a cutoff point of 15% of total pancreatic fat was significantly correlated with NAFLD diagnosis [64]. Decreasing liver fat content by 13% during weight loss after a very low-calorie diet was responsible for remission of T2D and a restoration of β-cell functioning in a group of responder patients [65].

### 3.3. T2D Develops When Hepatic Autoregulation Is Lost—A Matter of Fat and Diet

As described in detail in previous paragraphs, NAFLD incidence is closely related to the metabolism of FAs due to increased de novo lipogenesis (DNL), adipose tissue dysfunction, and decreased β-oxidation of fatty acids, which lead to mitochondrial failure, degeneration and inflammatory infiltration of hepatocytes [66]. When periods of overnutrition are sustained, abnormal production of adipocytokines (e.g., leptin, adiponectin, and TNF-α) or hepatokines with diabetogenic properties (e.g., fetuins, RBP4, and selenoprotein P) intensify peripheral insulin resistance. In turn, insulin resistance governs peripheral lipolysis by increasing the hepatic influx of FAs and initiation of NAFLD [58]. The biological mechanisms underlying the liver response upon exposure to elevated concentrations of FAs require further study. Nonparenchymal hepatic cell types, including hepatic stellate cells (HSCs), contribute to coordinated fibrosis and progression towards NAFLD [67]. High levels of saturated FAs from the diet lead to the activation of HSCs, which, in turn, upregulate the production of proinflammatory cytokines (IL-34 and CCL5) [67,68]. Through lipid droplet breakage, HSCs remain a source of bioactive lipid species that may act extracellularly within the liver, enhance the lipotoxic effect of fat spillover and lead to a more severe spectrum of NAFLD [69].

Type 2 diabetic patients with confirmed NAFLD exhibited lower values of homeostatic model assessment for β-cell function (HOMA-β) and reduced β-cell function, and had significantly more hepatic and adipocyte insulin resistance than their diabetic counterparts without NAFLD [70,71]. Furthermore, in a Finnish cohort, intrahepatic fat content shared the strongest correlation with fasting serum insulin and C-peptide [72]. A relative defect in β-cell function was observed in older individuals with NAFLD, who had lower insulin sensitivity and hepatic insulin production and increased C-peptide [73]. More recently, in a cross-sectional analysis of young Chinese adults, increased levels of ALT and γ-glutamyltransferase in T2D individuals were associated with the attenuation of β-cell function [74]. Importantly, the role of pancreatic fat should not be excluded from a discussion of the relationship between NAFLD and β-cell function. Dysfunction of β-cells per se is not considered a complication of NAFLD; however, accumulation of ectopic fat in the pancreas drives β-cell demise [73,75].

Interestingly, serum phospholipid ω-3 polyunsaturated fatty acid (PUFA) levels were significantly lower in patients with T2D complicated by NAFLD, and they were negatively correlated with insulin resistance [76]. Serum phospholipid FA abundance can reflect individual dietary FA intake [76]. Furthermore, recent studies revealed that dietary supplementation of ω-3 PUFA is potent in reducing hepatic steatosis, improving insulin resistance and ameliorating inflammation in NAFLD-affected individuals [77]. In fact, dietary docosahexaenoic acid (DHA) was the most effective PUFA in preventing the progression of hepatic fat accumulation and reducing fasting hyperinsulinemia in NAFLD patients and fa/fa Zucker diabetic rats with hepatic steatosis and diabetes [78,79]. This area of research is still very limited; however, only hypocaloric diets with either low-fat or low-carbohydrate energy deficits attenuated T2D development in patients with NAFLD [80,81].

Ultimately, NAFLD coexists with T2D and works synchronously to have reciprocal clinical outcomes. The presence of NAFLD increases the onset of T2D. Meanwhile, T2D is potent in accelerating the progression of NAFLD to more severe forms, including cirrhosis or hepatocellular carcinoma [58]. Therefore, screening for novel biomarkers and careful treatment recommendations for patients with T2D are justified and need to be actively pursued in the clinic. On the other hand, prompt diagnosis and management of abnormal fatty liver parameters will assist in minimizing liver-derived morbidity and mortality within diabetic cohorts.

## 4. Molecular Mechanisms of Hepatic Lipid Accumulation in NAFLD

The pathophysiology of NAFLD is mainly characterized by an accumulation of lipids [82]. Hepatic steatosis results from an imbalance between lipid acquisition and lipid clearance, which are regulated through four major pathways: (1) uptake of circulating lipids, (2) de novo lipogenesis (DNL), (3) fatty acid oxidation (FAO), and (4) export of lipids in VLDL [83]. In NAFLD, hepatic FA uptake and DNL are increased, while a compensatory elevation of FAO is insufficient to normalize lipid levels [83]. To establish the relative contribution of lipid accumulation in patients with NAFLD, stable isotope tracers were used. The study demonstrated that approximately 60% of liver triglycerides (TG) content was derived from free fatty acids (FFA) influx from adipose tissue, 26% from DNL, and 15% from diet [17].

### 4.1. Fatty Acid Uptake

Transmembrane FFA uptake in the liver is mainly due to the presence of plasma membrane transporters, such as fatty acid transport proteins (FATP2, FATP5), fatty acid translocase (CD 36), and fatty acid binding protein (FABP) [84]. Increased uptake of plasma FFAs derived from lipolysis in adipose tissue significantly contributes to NAFLD development, as studies of liver-specific knockout of FATP2 and FATP5 have established decreased FA uptake and hepatic steatosis [85,86], whereas liver-specific overexpression of fatty acid translocase (FAT/CD36) aggravates the condition [86]. Moreover, NAFLD and NASH patients show CD36 [87,88] and NASH patients exhibit FATP2 and 5 [87] gene upregulation in the liver, suggesting that these transporters contribute to hepatic steatosis and progression of fibrosis. Interestingly, no difference in hepatic FATP5 gene expression between individuals with and without hepatic steatosis was found [89]. Genetic variation has been suggested to underlie part of the contribution of FATP5 in NAFLD as a FATP5 promoter polymorphism (rs56225452), representing a putative gain-of-function mutation in the FATP5 promoter, and may be correlated with BMI-dependent hepatic steatosis in males with NAFLD [90].

Following uptake, hydrophobic FAs are transported between different organelles by FABP, of which, FABP1 is the predominant isoform in the liver [84]. In individuals with NAFLD, hepatic FABP1, FABP4, and FABP5 mRNA levels were increased compared to non-NAFLD controls, and FABP4 and FABP5 expression were correlated with the percentage of fat in the liver [89,91]. Thus, elevated intracellular trafficking of FA in the lipid-laden liver of NAFLD patients may be shunting harmful FAs to storage, thereby promoting steatosis [83].

### 4.2. Hepatic De Novo Lipogenesis

Hepatic DNL has been demonstrated to play a significant role in NAFLD pathogenesis and is increased in individuals with NAFLD [92]. The high rate of lipogenesis observed in hepatic steatosis is associated with hyperglycemia and hyperinsulinemia [93,94]. The induction of lipogenic genes is under the combined actions of sterol regulatory element binding protein-1c (SREBP1c) in response to insulin and carbohydrate responsive element binding protein (ChREBP) in response to glucose [95,96]. ChREBP was downregulated in patients with NAFLD compared to healthy controls, and instead, SREBP1c was shown to be one of the predominant regulators of DNL in NAFLD [97]. In response to elevated SREBP1c, the expression of downstream targets acetyl-CoA carboxylase (ACC) and fatty acid synthase (FAS) was increased in both patients with NAFLD and animal models of the disease [97,98,99].

The use of genetically engineered mice has helped to clarify that knockdown of enzymes involved in FA synthesis was able to reverse NAFLD [95]. ACC catalyzes the committed step of the de novo FA biosynthesis pathway by converting acetyl-CoA to malonyl-CoA. Two ACC isoforms (ACC1 and ACC2) were identified in animals [100]. Liver-specific knockout of ACC1 decreased hepatic lipid accumulation in mice and DNL in hepatocytes [101]. However, knockout mice were not protected from hepatic steatosis induced by a high-fat high-carbohydrate diet, which was accompanied by increased blood glucose, insulin, and FFAs [101]. Moreover, some but not all studies have suggested that global ACC2 knockout mice are protected against the development of obesity, diabetes, and NAFLD when fed a high-fat high-carbohydrate diet [102]. Consequently, inhibition of both ACC1 and ACC2 was required to improve hepatic steatosis in mice, implying that both isoforms are important in NAFLD [102].

Fatty acid synthase (FAS) catalyzes the de novo synthesis of FAs, thus determining the rate of hepatic DNL. Interestingly, liver-specific FAS knockout promoted hepatic steatosis in mice on a zero-fat diet, in which steatosis developed alongside defective peroxisome proliferator-activated receptor (PPAR)-α signaling [103].

Stearoyl-CoA desaturase 1 (SCD1) is the rate-limiting enzyme catalyzing the biosynthesis of monounsaturated fatty acids [104]. Mice with global knockout of SCD1 are protected from high-carbohydrate high-fat diet-induced obesity and show decreased lipogenic gene expression coupled with increased β-oxidation in the liver [105,106]. Accordingly, antisense nucleotide inhibitors against hepatic SCD1 also prevent high-carbohydrate high-fat diet-induced steatosis [107]. Interestingly, liver-specific SCD1 knockout mice are protected from obesity and hepatic steatosis induced by a high-carbohydrate diet, but not from hepatic steatosis induced by a high-fat diet [108]. Despite preventing steatosis, SCD1 knockout exacerbated hepatic fibrosis and cellular apoptosis in mice with NASH induced by an methionine- and choline-deficient (MCD) diet [109]. The end result of SCD1 downregulation may, therefore, be an exacerbation of NASH due to intracellular accumulation of cytotoxic saturated FAs [110]. Saturated FAs have been demonstrated to cause liver dysfunction by promoting endoplasmic reticulum (ER) stress and apoptosis [111,112]. In contrast to saturated FAs, an increase in monounsaturated FAs induced steatotic liver but did not initiate apoptosis [113].

Elongation of very long-chain fatty acids protein 6 (ELOVL6) is a microsomal enzyme that regulates the elongation of C12–C16 saturated FAs and monounsaturated FAs. The results from mouse models with loss or gain of function of ELOVL6 showed that this enzyme is crucial for the development of hepatic steatosis and liver injury, suggesting that the hepatic long-chain FA composition is a determinant in NASH [114].

The esterification of a fatty acyl moiety to diacylglycerol to form TGs is catalyzed by two diacylglycerol O-acyltransferases (DGATs). Inactivation of the hepatic DGAT2 isoform in obese mice results in a significant reduction in TGs but an increase in oxidative stress and hepatocellular apoptosis and a worsening of hepatic inflammatory activity and fibrosis. This observation suggests a protective role of TG against the development of hepatic inflammation [115] and is of particular interest because the hepatic concentration of diacylglycerols is increased in patients with NALFD [116].

### 4.3. Fatty Acid Oxidation and VLDL Secretion

A decrease in FAO, as well as a strong decrease in mitochondrial respiratory efficiency, was observed in animals fed a high-fat (HF) diet [95]. This last observation suggests that excess fat in the diet induces a partial uncoupling between respiration and phosphorylation in the mitochondria [117]. Vial et al. [117] hypothesized that less fatty acid oxidation (despite a higher capacity) could be related to inhibited oxidative phosphorylation (OXPHOS) at Complex IV and a lower cellular redox state with increased mitochondrial reactive oxygen species (ROS) production. On the other hand, studies of FAO in patients with or NASH are conflicting, reporting enhanced [118,119,120], unchanged [121], or decreased FAO [122]. The expression of genes related to mitochondrial and peroxisomal β-oxidation was higher in patients with more severe steatosis than in individuals with less severe steatosis or in nonsteatotic controls [123]. Additionally, β-oxidation, measured indirectly as plasma β-hydroxybutyrate levels, was higher in patients with NASH than in those with steatosis or normal controls [120].

Fatty acid oxidation is transcriptionally regulated by PPARα. In humans, hepatic PPARα levels did not differ between patients with steatosis and healthy individuals [89]. However, PPARα was downregulated in patients with NASH compared to both patients with steatosis and healthy controls [124,125], and the expression of PPARα decreases with increasing NAFLD activity score and progression of fibrosis [124]. In addition to FAO, another method of decreasing hepatic lipid content is exporting TG from the liver after packaging it into water-soluble VLDL particles alongside cholesterol, phospholipids, and apolipoproteins [126]. VLDL secretion was increased in patients with NAFLD, and hepatic TG content was directly associated with VLDL-TG secretion rates [126,127,128]. However, while VLDL-TG export increased with intrahepatic lipid content, secretion plateaued when fat content in the liver exceeded 10%, surpassing the compensatory capacity to prevent increasing hepatic lipid accumulation [126].

## 5. Dietary Rodent Models of NAFLD and NASH

Animal models are very useful in revealing the mechanisms involved in the pathogenesis of NAFLD progression. A growing number of animal models and NAFLD-inducing diets are available in the literature, enabling detailed studies of NAFLD and its progression to the more severe stages. It is necessary to remember that dietary NAFLD models focus on the metabolic situation in patients but may differ regarding clinical or morphologic aspects.

### 5.1. Choline-Deficient (CD) Diets

Choline is an essential nutrient that serves as a molecular building block for phospholipids, especially phosphatidylcholine (PC), or as a donor of methyl groups in S-adenosylmethionine production, and is thereby involved in mitochondrial bioenergetics and β-oxidation as well as VLDL production and secretion by hepatocytes [129,130,131,132]. CD diets induce a significant increase in liver triglycerides, leading to liver steatosis a few weeks after the initiation of the diet in mice and rats [133,134]. A recent study demonstrated moderate periportal micro- and macrovesicular liver steatosis in rats as early as 4 weeks after initiation of diet feeding, which could be further worsened by a prolonged feeding time of up to 12 weeks [135]. Long-term feeding of a CD diet for up to 52 weeks was shown to induce hepatocarcinogenesis with low incidence (~15%) in rats [134,136,137]. However, the livers of CD diet-fed animals (up to 12 weeks) at best, show only slight signs of inflammation or fibrosis [135,138]. Furthermore, the metabolic phenotype of this model poorly reflects the situation in patients, as these animals do not show significantly increased weight gain compared to animals on control diets or those with insulin resistance or increased serum free fatty acid (FFA) levels [133,134,139]. However, a variety of modifications have been added to this model to improve its relevance.

### 5.2. Semisynthetic Choline-Deficient L-Amino Acid-Defined (CDAA) Diet

In the CDAA diet, proteins are substituted with an equivalent and corresponding mixture of L-amino acids in addition to the deficiency in choline. Short-term treatments for up to 12 weeks showed a significantly increased steatotic phenotype and liver triglyceride (TG) content in CDAA-fed rat compared to the CD control group [140]. An increased feeding time of up to 22 weeks led to inflammation and pronounced fibrosis in mice [141]. Mice also showed increased body weight, plasma triglycerides and insulin resistance after 22 weeks of a CDAA diet [141,142]. The combination of a CDAA diet with a fat-enriched diet worsened the fibrotic NASH phenotype (6–9 weeks) in mice. However, metabolic alterations such as increased visceral fat depots or insulin resistance were not found in this model [143].

### 5.3. Methionine- and Choline-Deficient (MCD) Diet

The MCD diet is one of the most popular nutritional NAFLD, or more precisely NASH, models: the addition of methionine deficiency induced rapid and severe lobular inflammation and hepatocyte ballooning (after 2–8 weeks) and early-onset fibrosis (after 8–10 weeks) in C57BL/6 mice [144]. Liver damage was mirrored by increased serum aspartate aminotransferase (AST) and alanine aminotransferase (ALT) levels as early as 2 weeks after diet initiation that progressively increased [144], whereas the severity depended on species, strain, sex and duration of feeding [143,144,145,146,147,148]. The morphological characteristics of MCD-induced NASH include macrovesicular steatosis, perisinusoidal fibrosis, hepatocyte ballooning, apoptosis and necroinflammation (increase in proinflammatory and profibrogenic cytokine levels), as well as mitochondrial anomalies [145]. Similar to CD diets, the addition of a high-fat (HF) diet to the MCD diet accelerates the occurrence of the observed phenotype: extensive steatohepatitis with macro- and microvesicular steatosis and inflammatory foci can be observed in mice as early as 17 days after feeding [149]. However, the time of onset of fibrosis (~70 days after the beginning of feeding) does not differ from that of the normal MCD diet. In addition, MCD-induced weight loss in mice also persists under a HF diet [150,151]. Further studies in mice on an MCD-HF diet showed that at least 0.2% methionine supplementation is necessary to maintain body weight during treatment. The addition of more than 0.4% methionine to the diet led to the same weight gain observed in the pure HF diet. Unfortunately, supplementation with methionine above 0.2% does not result in an inflammatory or a fibrotic NASH phenotype [152]. The MCD diet model is a rapid and reproducible model of a NASH liver phenotype, but it does not exhibit any of the metabolic features of human NAFLD, including obesity, insulin resistance or dyslipidemia. Animals fed an MCD diet suffer from pronounced weight loss (up to 40% in 10 weeks), have low fasting blood sugar, peripheral insulin sensitivity, low serum insulin and decreased blood triglyceride and cholesterol levels [144,147,148,153]. Therefore, the MCD diet model is typically considered to be a model of fibrosating steatohepatitis but not a model that strictly reflects NAFLD.

### 5.4. High-Fat (HF) Diets

HF diets may be diverse but provide 45%–75% of the caloric intake as fat [145,147,154]. These diets cause an excess intake of FFAs, as well as increased lipolysis, leading to TG accumulation in hepatocytes [147]. A major advantage of these models is their high similarity to the metabolic profile of the human disease: obesity, insulin resistance and increased serum FFA and liver TG content that are found beginning at 10 weeks after feeding a HF diet in mice and rats [147,155,156]. However, progression of the disease can be observed only after extensive feeding (>34 weeks), with less pronounced signs of inflammation than, for example, those observed in the MCD diet model. A further extension of feeding by up to 50 weeks may lead to increased inflammatory infiltration, but to only minimal fibrosis [156]. Thus, pure HF diets are capable of recapitulating the metabolic features of human NAFLD but fail to represent disease progression towards a severe NASH phenotype. Accordingly, morphological features of progressive disease such as MDBs are rarely reported in these models [145]. Notably, both metabolic abnormalities and the NAFLD phenotype depend not only on species, strain and sex in terms of time of occurrence and degree but also, on the composition of dietary fats included in the diet. Compared to a standard HF diet, animals fed a trans-fat-enriched HF diet showed less weight gain but also developed more pronounced steatosis and liver damage after 8–16 weeks [157]. Feeding a trans-fat-enriched HF diet to mice significantly increased insulin resistance compared to a standard HF diet, although no differences in serum hyperlipidemia were found [157]. In rats, more severe steatosis was induced after 13 weeks of a trans-fat-enriched HF diet, accompanied by significantly increased insulin resistance [158]. In this study, animals fed a trans-fat-enriched HF diet developed more pronounced lipid profile disorders than animals fed a standard HF diet but showed no difference in liver damage [158]. Differences between saturated and unsaturated FAs were also described. Feeding rats a HF diet enriched with polyunsaturated fatty acids (PUFA) for 6 weeks resulted in obesity and increases in liver damage and serum sugar levels compared to rats fed an almost isocaloric control diet without the increased fat content. However, compared to rats fed a saturated fatty acid (SFA)-enriched HF diet, PUFA-fed animals showed significantly less weight gain and liver damage. Despite elevated serum glucose levels, the PUFA-enriched HF diet did not result in insulin resistance or an increase in serum insulin, as observed in animals in the SFA-rich HF diet group [159].

### 5.5. CD-HF Diet

Combining a HF diet (fat content 45 kcal% or higher) with choline deficiency worsened the CD diet model regarding its metabolic phenotype: HF diet feeding over 8 weeks in mice induced gains in body weight, adipose tissue depot weights and liver triglyceride levels. The addition of choline deficiency for the last two weeks of a HF diet feeding further increased liver triglyceride levels but did not further alter body weight gain or adipose tissue weight [139]. In this model, an 8-week HF diet induced insulin resistance that was significantly enhanced when the diet was changed to a CD-HF diet for the last two weeks of the diet [139]. In contrast, a study by Wolf et al. reported insulin resistance comparable to that of a HF diet but only after 6 months of continuous CD-HF diet feeding in mice [160]. Here, pronounced inflammation was observed after 6 months. Furthermore, signs of NASH (ballooned hepatocytes, satellitosis (i.e., abnormal cell clustering), MDB formation and glycogenated nuclei were observed after 12 months. Long-term CD-HF diet feeding also led to hepatocarcinogenesis after similar periods (~12 months) reported for the CD diet. The 25% tumor frequencies reported for the CD-HF diet model are higher than those reported for the CD diet (~15%), which may be due to differences in species (CD diet in rats [134,136,137] vs CD-HF diet in mice [160]) in the respective studies [134,136,137,160].

### 5.6. Western Diets

So-called “Western diets (WDs)” resemble Western dietary habits with a high concentration of saturated fats and simple carbohydrates. A high intake of simple carbohydrates alone is able to cause obesity and NAFLD in humans. The sole administration of fructose, sucrose or glucose (the most common simple carbohydrate sources), either through diet or drinking water, has been shown to trigger steatosis in rodents [161,162,163]. Fructose, in particular, is known for its lipogenic properties and for the aggravation of glucose and fat metabolism disorders, leading to increased visceral fat deposition, liver TG accumulation and insulin resistance [92,164]. The combination of fructose and a HF diet induced an increase in weight gain and steatosis in mice after 8 weeks of feeding compared to a pure HF diet. An extension of feeding up to 16 weeks with this combined diet resulted in significant inflammation in the liver but not in an increase in liver damage [165,166]. Similar results were found with a 15-week sucrose-supplemented HF diet: it increased body weight gain, liver TG values, steatosis values and TNF-a values compared to the pure HF diet. In this case, however, increased AST and ALT values were found in serum, indicating increased liver damage [167]. Rats developed insulin resistance as early as 2 weeks after starting to consume a fructose-supplemented HF (HFHF) diet. The HFHF diet group also showed a higher degree of steatosis than the HF diet group [168]. In a recent study, an HF sucrose pellet diet was administered to rats for 16 weeks, inducing significantly more pronounced steatosis, an increase in liver triglycerides and obesity compared to a pure HF diet treatment [169]. Surprisingly, the HF diet group in this study showed no increase in body weight or white fat deposits compared to the control diet group. The authors refer to a study by Sampey et al. on this point, which showed that rats fed an HF diet had a similar weight profile to rats fed an adjusted low-fat diet but gained more weight than rats fed a standard commercial chow, underlining the importance of choosing the right control diet [170].

The “American Lifestyle-Induced Obesity Syndrome” (ALIOS) diet published by Tetri et al. takes into account the influence of dietary fat composition on the NAFLD phenotype. This model uses a combination of a HF diet (45 kcal% with 30% fat content from trans fatty acids) and fructose-containing drinking water. This scheme leads to significant steatosis, inflammation and liver damage after 16 weeks of feeding. Glucose tolerance was reduced in the ALIOS model within 2 weeks, and after 4 weeks, fasting glucose levels were significantly higher than those in the control group. The fibrogenic response in the liver was detected at the molecular level, but after 16 weeks of feeding, no fibrosis was observed in liver histology. It is important to note that the steatotic and inflammatory liver phenotype was considerably improved by replacing the trans-fat content with standard fat (lard) [171].

Another model, the “fast-food mouse” developed by Charlton et al., adds 2% cholesterol to a WD with high (saturated) fat content (40 kcal% of which 12% is saturated) and fructose supplementation in the drinking water. The animals treated with this diet showed a weight gain comparable to that of HF diet-fed animals and developed insulin resistance [172].

Supplementation of cholesterol and cholate, also known as an “atherogenic diet”, leads to a NASH-like liver phenotype in rodents. However, as with the MCD diet model, this treatment results in weight loss, increased insulin sensitivity and lowered serum TG levels—a metabolic phenotype opposite to that of the human NAFLD/NASH situation [145]. A combination of the cholesterol/cholate treatment with a HF diet induced human NASH-like morphology (including MDBs and ballooned hepatocytes) with an even faster occurrence (after 12 weeks instead of 24 weeks) and without increased insulin sensitivity [173,174].

Charlton’s fast food model also demonstrated signs of NASH, including hepatocyte ballooning and lobular inflammation. However, even after 26 weeks of feeding, only mild fibrosis was observed in this model [172]. Similar results have been obtained with the Amylin liver NASH (AMLN) model [175,176,177]. Comparable to the “fast-food mouse” model, this diet is based on a HF diet but has a high trans-fat content (40 kcal% of which 18% is trans-fat) and 2% cholesterol, as well as high-fructose supplementation (20%) administered directly via the feed instead of via the drinking water [175,176,177]. 

In both models—Charlton’s “fast food mouse” and the AMLN diet—a promising candidate for the treatment of NASH was tested: Obeticholic acid (OCA). Diet supplementation with OCA over a period of 25 weeks in the “fast-food mouse” diet model significantly reduced the degree of liver steatosis, liver cholesterol content and hepatic insulin receptor signaling [178]. In the AMLN model, OCA supplementation also significantly improved the steatotic phenotype and liver triglyceride content. In addition, the administration of OCA reduced collagen and galectin-3 protein levels in the liver, both markers of fibrosis. However, no significant improvement in the degree of fibrosis under OCA supplementation was observed in the histological evaluation [179]. Comparable results were obtained from human NASH patients by the FLINT study [180]. The main result of this study was a decrease in NAFLD activity scores without worsening fibrosis by OCA treatment. This shows that dietary NAFLD models are suitable for studying the pathomechanisms in human patients. As models, however, they can only reflect specific aspects. Selection of such a model must, therefore, always be based on the underlying research question. Table 1 gives a brief overview of the dietary models described here and their most important properties in relation to liver and metabolic phenotypes.

## 6. The Effect of NAFLD-Inducing Diets on Mitochondrial Functioning

Mitochondria are known as the powerhouse of the cell due to their major role in adenosine triphosphate (ATP) production through the oxidative phosphorylation process. Nevertheless, this organelle is also critical to the control of lipid and carbohydrate homeostasis ([181] for a detailed review of the mitochondrial role in cellular metabolism). In the liver, FAs generated from the hydrolysis of intestinal chylomicrons and taken up from circulation due to lipolysis in white adipose tissue (WAT) or generated by DNL can be esterified into TGs or can enter mitochondria. While short-chain fatty acids (SCFAs) and medium-chain fatty acids (MCFAs) can freely enter mitochondria, the uptake of long-chain fatty acids (LCFAs) is mediated by carnitine palmitoyltransferase 1 (CPT1) [182].

In a fed state, high levels of insulin favor hepatic DNL and esterification of FAs into TAG, which can accumulate in the form of lipid droplets or can be secreted into the bloodstream and distributed throughout the body in the form of VLDL. In a fasting state, high levels of glucagon favor the transport of FAs into the liver, while decreased levels of malonyl-Coa (a metabolite generated during DNL that inhibits CPT1 in a fed state) facilitates FA transport into the mitochondria to undergo mitochondrial fatty acid β-oxidation (mtFAO) [183]. mtFAO generates acetyl-CoA molecules that can enter the tricarboxylic acid (TCA) cycle or undergo ketogenesis under low glucose availability conditions. In the latter condition, ketone bodies (acetoactetate and β-hydroxybutyrate) are mobilized to extrahepatic peripheral tissues where they are oxidized by the TCA cycle for ATP synthesis [184]. As mentioned above, mtFAO and the TCA cycle can generate reducing equivalents of nicotinamide adenine dinucleotide (NADH) and flavin adenine dinucleotide (FADH_2_), which are reoxidized in the electron transport chain (ETC) of the mitochondria by a process coupled to the synthesis of ATP. During this process, electrons are transferred along mitochondrial complexes in the inner mitochondrial membrane (IMM) until they associate with protons and oxygen to produce water. Nevertheless, a fraction of the electrons could leak mostly from Complex I and Complex III of the ETC, contributing to superoxide anion radical production [185]. Other mitochondrial enzymes were also recently reported to contribute to the production of ROS [186]. Under normal conditions, mitochondria efficiently quench ROS production via their mitochondrial antioxidant defenses (Mn superoxide dismutase (MnSOD), Cu/Zn superoxide dismutase (Cu/ZnSOD), catalase (CAT), glutathione (GSH) and thioredoxin 2 (TRX2) systems), and residual ROS are considered important signaling molecules [187]. However, an imbalance between ROS species and antioxidant defenses has been reported to trigger oxidative stress and tissue damage [188,189].

### 6.1. Mitochondrial Metabolic Dysfunction in NAFLD

Alterations in the structure of mitochondria along with changes in mitochondrial metabolism have been reported in several models of NAFLD [165,190,191]. Although the mechanisms of NAFLD development and progression are still not completely understood, a causative link has been addressed between mitochondrial dysfunction and exacerbated oxidative stress in the pathophysiology of steatosis and progression to NASH [192]. Dietary intervention has been implemented as the best approach to mimic the human features of NAFLD in in vivo models; however, the percentage of fat as well as its composition widely differ between different studies. HF diets include compositions from 13% to 71% of total calories in terms of fat, with the origin of fat predominantly based on lard and vegetable oils [190,193]. However, most of the authors fail to include a detailed composition of the diet. Taking that into account, in the current section, there is an analysis of the metabolic mitochondrial changes promoted by different diets in a NAFLD context.

### 6.2. Similarities and Controversies Concerning the Effect of NAFLD-inducing Diets on Mitochondria

The bioenergetics of mitochondria varied significantly within different NAFLD animal models under distinct dietary interventions (Table 2). 

Therefore, a decrease in respiratory capacity using glutamate/malate and pyruvate/malate as Complex I substrates or succinate plus rotenone as Complex II substrates has mostly been reported [117,159,200]. Nevertheless, the same trend was observed with the use of palmitoyl-carnitine as a lipid substrate in combination with malate [197,199]. However, a few contradictory studies have described an increase in mitochondrial respiration in the presence of the same mitochondrial substrates mentioned previously, namely, palmitoyl-carnitine [159,191,201,205]. Those contradictory results were obtained with the use of diets in which 60% of the total calories were from fat, and the fat source was based on lard and soybean oil. However, these discrepancies cannot be explained by the dietary intervention chosen, since the same diet has been shown to decrease respiration, as stated in Table 2. In agreement with a decreased mitochondrial respiratory capacity in steatotic livers, decreased OXPHOS activity was reported by different authors [117,196,198,199,202]. A high-lard diet supplemented with cholesterol induced a significant reduction in Complex IV activity after a short 8-week treatment in mice [190]. This decrease was further supported by data from longer studies. Nigro et al. observed a decrease in Complex I activity using a HF diet supplemented with fructose [196]. Similar findings were observed when fructose was replaced by sucrose as a supplement to a HF diet [203]. Fructose may act as an inducer of a more aggressive NAFLD phenotype compared to the effect of a HF diet alone [196]. A decrease of 50%–60% in the activity and assembly of all OXPHOS complexes has been observed with the progression of NAFLD to NASH [202], which may be justified by a marked reduction in OXPHOS subunit expression, particularly in mitochondrial DNA-encoded subunits. Interestingly, this process is correlated with some findings describing a decrease in mtDNA in the liver of mice fed a HF diet supplemented with sucrose [203,204]. However, it should be highlighted that other diets lacking sucrose and mostly composed of lard did not show the same pattern regarding the depletion of mtDNA levels in steatotic mice and rats [199,201]. Interestingly, there is also evidence that a reduced amount and activity of OXPHOS complexes may be explained by ROS-associated oxidative damage. Although ROS may generate lipid- and protein-oxidized species, ROS are also involved in the generation of 8-hydroxydeoxyguanosine (8-OHdG), a DNA oxidative marker found in the liver of NASH mouse models [202,206]. Therefore, a decrease in respiration and OXPHOS activity and expression could be linked to the accumulation of nDNA and mtDNA lesions.

It is widely accepted that an initial compensatory mechanism could protect the organism against nutrient overload intake and weight gain during the development of fatty liver [207]. Chan et al. reported an initial upregulation of genes involved in mtFAO (e.g., *Cpt1l*, *Pparα*, and *Crat*) in mice fed a HF diet supplemented with sucrose and cholesterol for 4 weeks [208]. Although this compensation is abolished at 10 weeks of feeding, upregulation of lipogenic and cholesterol metabolism genes occurred at this timepoint. This study is in agreement with several other studies showing an increase in mtFAO and stimulation of the TCA cycle, mostly in models fed lard-based diets [191,194,201,209]. Notably, Franko et al. showed increased levels of proteins involved in mtFAO but without any changes in respiratory capacity or in the expression and levels of OXPHOS complexes after 20 weeks of HF diet feeding [210]. One paper showed a decline in FAO in a NASH rat model fed a diet composed of lard and cholesterol [211]. These studies support the idea that early adaptations could contribute to the stabilization of energy homeostasis in steatotic stages, although data indicate that this state cannot be sustained during NASH progression.

Importantly, Lionetti et al. suggested that the effects of the fat-enriched diets on steatosis and mitochondrial function in a NAFLD phenotype may differ based on the composition of the diets in terms of saturated and unsaturated fatty acids [159]. This author has shown that a high-lard diet (high content of saturated fatty acids) induced lipid accumulation, mitochondrial dysfunction and oxidative stress after 6 weeks of treatment in rats. On the other hand, a diet enriched with fish oils (unsaturated fatty acids) is associated with better mitochondrial function and dynamics [159]. In contrast, dietary fructose was shown to strongly activate lipid accumulation, oxidative stress and inflammatory pathways compared to a high-saturated FA diet in mice after 12 weeks of feeding [196].

Overall, there is no consensus about the effects of fat- and carbohydrate-enriched diets on mitochondrial metabolism in NAFLD. This is possibly due to experimental variations in the different studies, which include different diet compositions and durations of treatment. As a result, different rodent phenotypes are obtained, making it difficult to compare and establish assumptions within the current literature.

### 6.3. Influence of NAFLD-Inducing Diets on Liver Mitochondrial Morphology

Mitochondria may exist as extensive tubular networks or as single organelles. Their current morphology is shaped by dynamic processes of fusion and fission that are highly sensitive towards metabolic alterations [212,213]. The morphology of mitochondria is strongly linked to mitochondrial bioenergetics, since the fusion of single mitochondria into larger networks is positively linked to increased ATP production, while inhibition of fusion is linked to impairment of OXPHOS, mtDNA depletion and ROS production. Furthermore, fusion and fission processes are also involved in mitochondrial quality control and thus, in mitochondrial life cycle control [214].

In NAFLD patients, the biogenesis of new mitochondria is inhibited and decreases as the disease progresses towards NASH, while the total mitochondrial mass of the liver increases [215]. This phenomenon indicates an accumulation of obsolete mitochondria and impaired mitochondrial quality control. Morphologically, mitochondria from NAFLD patients appear round and swollen and show a loss of cristae structure, while in more advanced disease states, crystalline structures and megamitochondria are found [120,216,217]. The observed morphological deformities and abnormal build-up and degradation processes indicate changes in mitochondrial dynamics during NAFLD in human patients. In this section, we provide a brief overview of the influence of different NAFLD-inducing diets on the morphology of liver mitochondria.

Mitochondria from animals fed a HF diet were described as rounder and shorter with morphological anomalies such as enlarged or missing cristae, signs of swelling and matrix condensation [159,218]. Interestingly, one study showed the complete absence of mitochondrial changes under a HF diet when animals were forced to undergo regular endurance training [219]. The observed structural changes indicate increased fission of mitochondria, and upregulation of fission proteins in the livers of HF diet-fed animals has been observed in several studies [159,218,220,221]. Ultrastructural electron microscopy (EM) analysis also revealed increased mitochondrial fission in the livers of HF diet-fed animals [220]. A blockade of mitochondrial fission by inducible ablation of DRP1 led to a massive reduction in HF diet-induced steatosis, liver damage and oxidative stress [221].

An exchange of the saturated fatty acids in the HF diet with unsaturated fatty acids led to a substantial improvement of the mitochondrial structure under the HF diet. This diet induced an increased occurrence of “boomerang-shaped” mitochondria and the formation of mitochondrial clusters in hepatocytes, a characteristic sign of the formation of a mitochondrial network [159,222]. Indeed, a significant reduction in the expression of fission proteins and an induction of fusion proteins was observed in the livers of these animals [159]. Enlarged cristae and rounded mitochondria were also observed in the liver cells of animals fed a WD [165]. In addition, Einer et al. found changes in mitochondrial lipid composition associated with increased fluidity of the IMM. No significant changes in protein content or composition of WD mitochondria were found. However, a recent study showed differences in mitochondrial protein turnover in the livers of WD-fed animals [223]. Significantly increased half-lives of proteins in the outer mitochondrial membrane were observed, while the half-lives of proteins from the matrix and IMM were significantly decreased. Studies on the expression of mitochondrial biogenesis markers in NAFLD models showed decreased expression with a HF diet model, as well as with a WD diet and an MCD-based NASH model, thus indicating a general suppression of mitochondrial biogenesis in steatosis [223,224,225]. However, since the restoration of biogenesis or expression of biogenesis markers could be achieved with completely different, independent intervention strategies in two of these models, the observed suppression of mitochondrial biogenesis is most likely mediated by different diet-specific mechanisms but not by a general NAFLD-related pathogenic mechanism [224,225].

In a recent study, a WD was applied in the LPP rat model of Wilson’s disease [226]. Wilson’s disease is a genetic disorder of copper homeostasis that is frequently associated with liver steatosis and is, therefore, often misdiagnosed as NAFLD [227,228]. Excessive copper accumulation in the liver mitochondria of Wilson patients leads to mitochondrial changes (altered cristae, disturbed bioenergetics) similar to those described in NAFLD patients [217,229]. Feeding a WD to the LPP rat led to an earlier onset of the disease at approximately 3 weeks and a significantly more severe course of the disease. Liver mitochondria of WD-fed LPP rats were severely affected, with separated inner and outer membranes, excessive matrix condensation and swollen cristae [226]. This study further underlines the important influence of dietary intake on liver mitochondrial morphology and function. A short overview of morphological changes in mitochondria with different dietary NAFLD models can be found in Table 3.

## 7. Oxidative Stress in NAFLD: Sources, Defenses and Comparative Study of Different Diets

Oxidative stress plays a pivotal role in the initiation of NAFLD and in its progression to NASH, even when the molecular mechanisms underlying NAFLD are not yet entirely known. The oxidative stress theory of NAFLD was first postulated by Day CP and James OF in the 1990s [12]. This study proposed steatosis as the first “hit” for the initiation of NAFLD. However, the progression of benign NAFLD to NASH requires a second hit, possibly involving excessive formation of ROS, which are capable of inducing oxidative stress [12,230]. Since then, the contribution of oxidative stress in NAFLD and NASH has been widely studied. A wide range of oxidative stress and antioxidant markers are used in the evaluation of NAFLD progression and severity. Here, we will briefly review the role of oxidative stress in NAFLD pathogenesis and the effect of different diets on oxidative stress and NAFLD development.

### 7.1. Sources of ROS in NAFLD

Although ROS are derived from diverse endogenous cellular sources, mitochondria are considered to be the major contributor to ROS production. The ER is also an important organelle that contributes to cellular ROS during protein folding. Moreover, there are enzymatic sources of free radicals in the cytosol. Normally, at moderate concentrations, ROS act as secondary messengers to maintain physiological functions. However, excessive generation of ROS disrupts redox homeostasis and leads to ROS-induced cell damage. Of particular significance for NAFLD, is the mitochondria. Impaired mitochondrial function and the subsequent onset of ROS production form a vicious cycle that has been proposed as the critical player in NAFLD progression [231]. Indeed, nonalcoholic steatohepatitis is currently considered a mitochondrial disease [232]. Other authors have previously reported that mitochondria-derived ROS can be increased due to increased electron transfer to the ETC from the oxidation of FAs [233]. Hepatic FA oxidation rates were shown to be augmented early in patients with NAFLD, triggering electron transfer along the ETC [234,235]. Additionally, defective hepatic oxidative phosphorylation in patients with NAFLD leads to electron leakage and a reduction in ATP synthesis, resulting in enhanced ROS production [236,237]. Thus, there is a correlation between the electrons that “escape” from the ETC and the generation of superoxide radical anions (O2^•−^). The main O2^•−^ source within mitochondria is found in Complexes I and III. ROS production by Complex I (NADH dehydrogenase) is mediated via electron leakage from the flavin moiety (FMN) [238]. Complex III (ubiquinol-cytochrome c oxidoreductase) also contributes to the main O2^•−^ generation through the Q_0_ site [239]. Reports support that ROS produced by Complexes I and III are released from the mitochondria to the cytosol through voltage-dependent anion channels (VDAC), causing cellular oxidative damage [240,241].

Abundant evidence indicates that the 66 kDa isoform of the growth factor adaptor Shc (p66Shc) contributes to liver fibrosis by mediating mitochondrial ROS production [242,243]. p66Shc is a redox enzyme that catalyzes the reduction of oxygen to hydrogen peroxide (H_2_O_2_) under stress conditions through the oxidation of cytochrome c [244]. The activation of the pro-oxidant p66Shc signaling pathway is implicated in the control of cellular oxidative damage and stress-induced apoptosis [245]. Inhibition of p66Shc has been recently noted to mitigate mitochondrial-derived ROS production, reducing liver damage and attenuating fibrosis development [242]. Apart from the sites of ROS generation along the ETC, there are other mitochondrial sources of ROS in NAFLD. Mitochondrial pyruvate dehydrogenase (PDH) and α-ketoglutarate dehydrogenase (α-KGDH) complexes of the TCA cycle are highly vulnerable to environmental changes. Both mitochondrial complexes are considered to be indirect sources of ROS. Upon increased NADPH levels, PDH and α-KGDH produce O2^•−^ and H_2_O_2,_ contributing to increased oxidative stress [246]. Moreover, the mitochondrial isoform of cytochrome P450 is also considered a key inducer of ROS during NAFLD development [247]. NASH patients showed high levels of mitochondrial cytochrome P450 activity, which is correlated with increased ROS production [248].

### 7.2. Antioxidant Defense in NAFLD

To counteract ROS overproduction, cells have evolved an antioxidant defense system, which includes enzymatic and nonenzymatic antioxidants. A large majority of the cellular antioxidants are enzymatic. Within the enzymatic defense system, cytosolic superoxide dismutase 1 (SOD1) and mitochondrial superoxide dismutase 2 (SOD2) catalyze the dismutation of O2^•−^ to H_2_O_2._ This reaction is coupled with enzymes that neutralize H_2_O_2,_ such as catalase (CAT) and the glutathione system. CAT is located in cellular sites of H_2_O_2_ generation, especially in the mitochondria and peroxisomes [249]. However, CAT is an enzyme that has low affinity for its substrate and is more efficient under high levels of ROS. The glutathione system is the main antioxidant defense under mild or low levels of oxidative stress. Among the nonenzymatic antioxidants, substances obtained from the diet (carotenoids, tocopherols, ascorbic acid, vitamin E, etc.) and natural molecules synthetized by the organism (uric acid, albumin, glutathione, bilirubin, melatonin, etc.) have received particular attention [250].

Based on recent clinical trials, several antioxidants have been identified as possible biomarkers for the evaluation of NAFLD. These studies show that NAFLD correlates with decreased enzymatic and nonenzymatic antioxidant defense. Patients with NAFLD and NASH exhibit lower levels of serum antioxidants, including the glutathione system and CAT and SOD enzymes, which, in turn, result in higher susceptibility to cellular oxidative damage [251,252,253]. In addition, ascorbic acid deficiency has been associated with the promotion of NAFLD, which suggests an association between the diet and development of this disease [254,255]. Levels of circulating tocopherol and carotenoids have also been found to be decreased in NASH patients [256]. Interestingly, antioxidant treatments based on the use of vitamin E or melatonin can increase the enzymatic antioxidant defense, showing favorable outcomes in hepatic inflammation and steatosis [256]. Therefore, the impaired antioxidant defense system seems to be a critical factor in the pathogenesis of NAFLD.

### 7.3. Dietary Patterns and Oxidative Stress in NAFLD

FAs are known to be risk factors for diabetes, obesity and metabolic syndrome [257,258]. There is emerging evidence that dietary FAs are also critical for the onset of NAFLD. Barr and colleagues characterized and compared the metabolic phenotype of the liver between control and NAFLD mice and found different FA profiles [259], emphasizing the importance of diet in the development of this pathology. Although fat deposition in the liver is associated with fatty liver disease, it is uncertain why not all patients with NAFLD develop more advanced disease. Oxidative stress is considered to be at the forefront of the transition from NAFLD to NASH. However, little is known about the role of FAs and the effect of different diets on the metabolic functions of the liver and on the oxidative stress response. In recent years, several diets have been used to generate mouse models for the study of the molecular basis of NAFLD and its progression to NASH, cirrhosis and hepatocellular carcinoma [148,260,261]. The evaluation of the oxidative stress response through the progression of the disease represents a challenge to merge features of NAFLD patients and mouse models and provide mechanistic insights and therapeutic opportunities (see Table 4).

Obesity has been widely studied in recent decades, and a close connection between excessive body weight and ROS overproduction has been established [272]. A high-fat low-carbohydrate diet leads to impaired mitochondrial function and increased cellular oxidative damage [273]. Notably, obesity is commonly associated with NAFLD. A HF diet and obesity cause a spectrum of liver abnormalities, including insulin resistance, steatosis and persistent inflammation [274,275]. Indeed, several studies have shown that a HF diet induces a hepatic steatosis profile that is characteristic of NAFLD in patients [276,277]. Zischka’s group showed that a HF diet (28% saturated, 57% monounsaturated fatty acids and 13% polyunsaturated fatty acids) impaired mitochondrial function, resulting in decreased ATP production and reduced calcium sensitivity in mice with diet-induced NAFLD. However, these metabolic alterations were not associated with increased ROS production [165]. Moreover, in a previous publication, the authors demonstrated that the mitochondrial antioxidant defense response displayed by glutathione peroxidase 1 and glutathione S-transferase enzymes was reduced in mice fed a HF diet [278]. Interestingly, Dhibi et al. reported that different types of dietary FAs induce diverse effects on NAFLD. Diets enriched in trans fatty acids exert harmful effects on the hepatic oxidative status through an enhancement of lipid peroxidation and a reduction in superoxide dismutase, catalase and glutathione peroxidase activities, leading to NAFLD development [262]. In another study on NAFLD mice, an association was found between a HF diet enriched with higher concentrations of the saturated fatty acids C14:0, C16:0 and C18:0 and NAFLD development [279]. Moreover, a lipid signature that positions C14:0, C16:0, C16:1n-7, C18:1n-7, C18:1n-9 and C18:2n-6 as the main lipids that exhibit an accumulation during the NAFLD/NASH transition has also been characterized in NASH patients [280]. The fatty acids C16 and C18 are involved in the modulation of mitochondrial function [281], and their abnormal accumulation preceded oxidative stress and lipotoxicity [263,264,282]. Furthermore, a high-fat and high-cholesterol diet potentially contributes to hepatic steatosis and oxidative stress [174,265,283]. Generally, a HF diet is associated with an altered cellular antioxidant defense that contributes to TNF-α-induced hepatotoxicity in NAFLD [284,285]. To a considerable extent, the importance of fatty acids lies in the ability of cell membranes to adapt their composition based on dietary FAs, influencing their vulnerability to cellular oxidative damage and the antioxidant response [262,286,287,288].

Increasing consideration has been given to the effect of certain nutrients on oxidative stress and NAFLD progression. A large number of experimental studies have investigated the impact of an MCD diet on NAFLD because these two amino acids are crucial for mitochondrial β-oxidation [289]. Under these conditions, a correlation between increased ROS and lipid peroxidation levels and the severity of steatohepatitis and fibrosis has been established [266,267,268,290]. Fructose and sucrose are also considered major mediators of NAFLD. A low-fat, fructose-rich diet results in marked oxidative stress levels in hepatocytes [269]. Moreover, García-Berumen et al. demonstrated that the supplementation of a HF diet with fructose induces more severe hepatic damage as shown by the inhibition of state 3 and the impairment of Complex I activity, which subsequently promotes further mitochondrial ROS production [270]. Recent work has pointed to the crucial role of fructose and its close relationship with sucrose in the development of NAFLD. Mice fed a high-fat, high-sucrose diet showed severe steatosis, increased mitochondrial oxidative stress and defective antioxidant defense. Curiously, fructokinase deficiency exhibited a protective effect in NAFLD progression, indicating that a HF diet enriched with sucrose induces steatohepatitis in a fructose-dependent manner [167]. This study emphasizes the importance of fructose in the development of NAFLD and NASH. In addition, high levels of serum ferritin are frequently observed in NAFLD patients, showing signs of hepatic iron overload [291]. A copper-deficient diet is paralleled by higher serum ferritin levels and iron homeostasis perturbations. A reduced liver copper concentration and an excess iron overload lead to increased inflammation and oxidative injury of the liver, which contribute to NAFLD development [271,292].

## 8. Conclusions

NAFLD is a common disease in Western society and ranges from steatosis to steatohepatitis to end-stage liver disease. The molecular mechanisms that cause the progression of steatosis to severe liver damage are not fully understood. Different animal models allow in-depth studies of the pathogenesis and progression of NAFLD. A considerable number of diets used to study NAFLD in rodents that have recently been developed are based on nutritional deficiencies (e.g., choline or methionine deficiency) or diets similar to the eating habits of Western society. Diet composition influences the phenotype and development of NAFLD and its transition to NASH. The outcomes derived from the diverse studies address the potential effect of certain fatty acids, fructose, sucrose and other nutrients on oxidative stress accompanying the progression of NAFLD to NASH and more severe stages of the disease.

## Figures and Tables

**Table 1:** Overview on phenotypes of different dietary nonalcoholic fatty liver disease (NAFLD) models.

Diet	Time of Onset	Liver Phenotype	Metabolic Phenotype	Animal Model	Reference
Choline-deficient diet	4–12 weeks	increased liver triglycerides ^#^	no increased weight gain	Rat, Mouse	[133,134,135,136,137,138,139]
		steatosis ^#^			
		no inflammation			
		no fibrosis			
	52 weeks	hepatocarcinogenesis (~15% incidence) ^#^		Rat	[134,136,137]
Choline-deficient high fat diet	31 weeks	steatosis ^#^	increased weight gain ^#^	Mouse	[160]
		inflammation ^#^	insulin resistance ^#^		
			serum dyslipidemia ^#^		
	52 weeks	signs of nonalcoholic steatohepatitis (NASH) (ballooning, mallory denk bodies (MDBs), satellitosis, glycogenated nuclei) ^#^			
		hepatocarcinogenesis (~25% incidence) ^#^			
Semisynthetic choline-deficient L-amino acid-defined diet	4–12 weeks	increased liver triglycerides ^#^	no increased weight gain	Rat	[140]
		steatosis ^#^			
	22 weeks	inflammation ^#^	increased weight gain ^#^	Mouse	[141,142,145]
		fibrosis ^#^	insulin resistance ^#^		
			serum dyslipidemia ^#^		
Semisynthetic choline-deficient L-amino acid-defined high fat diet	6–9 weeks	increased liver triglycerides ^#^	no increased weight gain	Mouse	[143]
		steatosis ^#^	no insulin resistance		
		inflammation ^#^			
		fibrosis ^#^			
Methionine- and choline deficient diet	2–8 weeks	steatosis ^#^	weight loss	Rat, Mouse	[143,144,145,146,147,148]
		inflammation ^#^	no insulin resistance		
			(low serum insulin)		
			no serum dyslipidemia:		
			(decreased serum triglycerides)		
			(decreased serum cholesterol)		
	8–10 weeks	signs of NASH (ballooning) ^#^			
		fibrosis ^#^			
Methionine- and choline deficient high fat diet	2 weeks	steatosis ^#^	weight loss	Mouse	[149,150,151,153]
		inflammation ^#^			
	10 weeks	fibrosis ^#^			
High fat diet	10 weeks	increased liver triglycerides ^#^	increased weight gain ^#^	Rat, Mouse	[145,147,154,155,156,157]
		steatosis ^#^	insulin resistance ^#^		
	>34 weeks	inflammation ^#^	serum dyslipidemia ^#^		
	50 weeks	no fibrosis ^#^			
High fat diet with later added choline deficiency	8 weeks	increased liver triglycerides ^#^	increased weight gain ^#^	Mouse	[139]
		steatosis ^#^	insulin resistance ^#^		
			increased adipose tissue depot ^#^		
Western diet	8 weeks	steatosis ^#^	increased weight gain ^#^	Mouse, Rat	[165,166,167,168,169,170,171]
	16 weeks	inflammation ^#^	insulin resistance ^#^		
		no fibrosis			
Cholesterol supplemented Western diet	26 weeks	steatosis ^#^	increased weight gain ^#^	Mouse	[172]
		inflammation ^#^	insulin resistance ^#^		
		fibrosis ^#^			
		signs of NASH (ballooning) ^#^			

Note: **^#^** indicates features that are similar in rodents and humans.

**Table 2:** Mitochondrial response to the NAFLD-inducing diets.

Mitochondrial Response	Alterations	Diet	Reference
Mitochondrial fatty acid β-oxidation (TCA cycle)/tricarboxylic acid (TCA) cycle	Induction	High fat (lard; soybean oil)	[191]
		High fat (lard)	[194]
		High fat (lard; soybean oil)	[195]
		High fructose	[196]
		High fat (lard or fish oil)	[159]
Respiratory activity	Reduction	High fat (palm oil)	[197]
		Choline deficient diet	[198]
		High fat (lard)	[199]
		High fat (lard or fish oil)	[159]
		High fat (butter (C16:0, C18:0))	[200]
		High fat (lard; soybean oil)	[117]
	Induction	High fat (lard; soybean oil)	[201]
		High fat (lard; soybean oil)	[191]
Oxidative phosphorylation (OXPHOS) complexes activity	Reduction	High fat	[202]
		High fat (lard; soybean oil) (45% saturated fatty acids (FAs))	[196]
		Choline deficient diet	[198]
		High fat (61% saturated FAs) and sucrose	[203]
	Maintenance	High fat (lard)	[199]
	Induction	High fat (lard; soybean oil)	[191]
		High fructose	[196]
Mitochondrial ATP production	Reduction	High fat (vegetable oil) and fructose and glucose	[165]
		High fat (lard; soybean oil)	[117]
	Induction	High fat (lard; soybean oil)	[191]
		High fat (lard)	[199]
Mitochondrial DNA	Reduction	High fat (61% saturated FAs) and sucrose	[203]
		High fat (coconut and soybean oil) and sucrose	[204]
	Maintenance	High fat (lard)	[199]
	Induction	High fat (lard; soybean oil)	[201]

**Table 3:** Overview on dietary NAFLD models and mitochondrial morphology and dynamics.

Dietary Model	Mitochondrial Morphology	Mitochondrial Dynamics	Animal Model	Reference
High fat diet	round and shortend	upregulation of fission proteins	Rat, Mouse	[159,218,219,220,221]
	signs of swelling			
	enlarged or missing cristae			
	matrix condensation			
High fat diet with unsaturated fatty acids	“boomerang shaped”	attenuation of fission protein expression	Rat	[159,225]
		induction of fusion proteins		
	formation of mitochondrial clusters	decreased biogenesis markers		
Western diet	rounded	increased half-life of outer mitochondrial membrane (OMM) proteins	Mouse	[165,223]
	enlarged cristae	decreased half-life of inner mitochondrial membrane (IMM) proteins		
	increased fluidity of the IMM	decreased biogenesis markers		
Methionine-choline deficient diet		decreased biogenesis markers	Mouse	[224]

**Table 4:** Mechanisms whereby different diets regulate oxidative stress and oxidative damage.

Oxidative Stress Oxidative Damage	Mechanism	Diet	Reference
Maintenance	Mitochondrial H_2_O_2_ production Aconitase activity Protein carbonylation	High-fat (28% saturated, 57% monounsaturated and 13% polyunsaturated fatty acids)	[165]
Induction	Lipid peroxidation	High-fat (trans-fatty acids)	[262]
	Lipid peroxidation	High-fat (C16 and C18)	[263] [264]
	Reactive oxygen species (ROS) production Lipid peroxidation Protein carbonylation	High-fat and high-cholesterol diet	[265] [174]
	Cellular ROS production Mitochondrial ROS production Lipid peroxidation	Methionine-choline deficient diet	[266] [267] [268]
	Lipid peroxidation Nitrotyrosine	High-fructose diet	[269]
	Mitochondrial ROS production Lipid peroxidation	High-fat and high-fructose diet	[270]
	Superoxide generation Mitochondrial NADPH oxidase 4 (NOX4)	High-fat and high-sucrose diet	[167]
	Lipid peroxidation	Copper deficient diet	[271]
